# Developmentally anomalous cerebellar encephalocele arising within the cerebellopontine angle and extending into the adjacent skull base in a pediatric patient

**DOI:** 10.1007/s00381-020-05020-8

**Published:** 2021-02-10

**Authors:** Forrest A. Hamrick, Michael Karsy, Carol S. Bruggers, Angelica R. Putnam, Gary L. Hedlund, Samuel H. Cheshier

**Affiliations:** 1 Division of Pediatric Neurosurgery, Department of Neurosurgery, University of Utah, 100 N. Mario Capecchi Drive, Salt Lake City, UT 84113 USA; 2 Primary Children’s Hospital, University of Utah, Salt Lake City, UT USA; 3 Division of Neuro-Oncology, Department of Pediatrics, University of Utah, Salt Lake City, UT USA; 4 Department of Pathology, University of Utah, Salt Lake City, UT USA; 5 Department of Medical Imaging, Primary Children’s Hospital, University of Utah, Salt Lake City, UT USA

**Keywords:** Heterotopia, Hamartoma, Atypical teratoid–rhabdoid tumor, Cerebellopontine angle, Internal auditory canal, Encephalocele

## Abstract

Lesions of the cerebellopontine angle (CPA) in young children are rare, with the most common being arachnoid cysts and epidermoid inclusion cysts. The authors report a case of an encephalocele containing heterotopic cerebellar tissue arising from the right middle cerebellar peduncle and filling the right internal acoustic canal in a 2-year-old female patient. Her initial presentation included a focal left 6th nerve palsy. Magnetic resonance imaging was suggestive of a high-grade tumor of the right CPA. The lesion was removed via a retrosigmoid approach, and histopathologic analysis revealed heterotopic atrophic cerebellar tissue. This report is the first description of a heterotopic cerebellar encephalocele within the CPA and temporal skull base of a pediatric patient.

## Introduction

Cerebellopontine angle (CPA) lesions are uncommon in children. Tumor mimickers in this region have been described, especially in cases of neurofibromatosis type 2, but often involve one type of tumor mistaken for another [1, 2]. Here, we describe a patient with left 6th cranial nerve (CN) palsy whose imaging workup revealed a right CPA encephalocele emanating from the middle cerebellar peduncle that was later identified as containing heterotopic cerebellar tissue.

## Case report

**Fig. 1:**
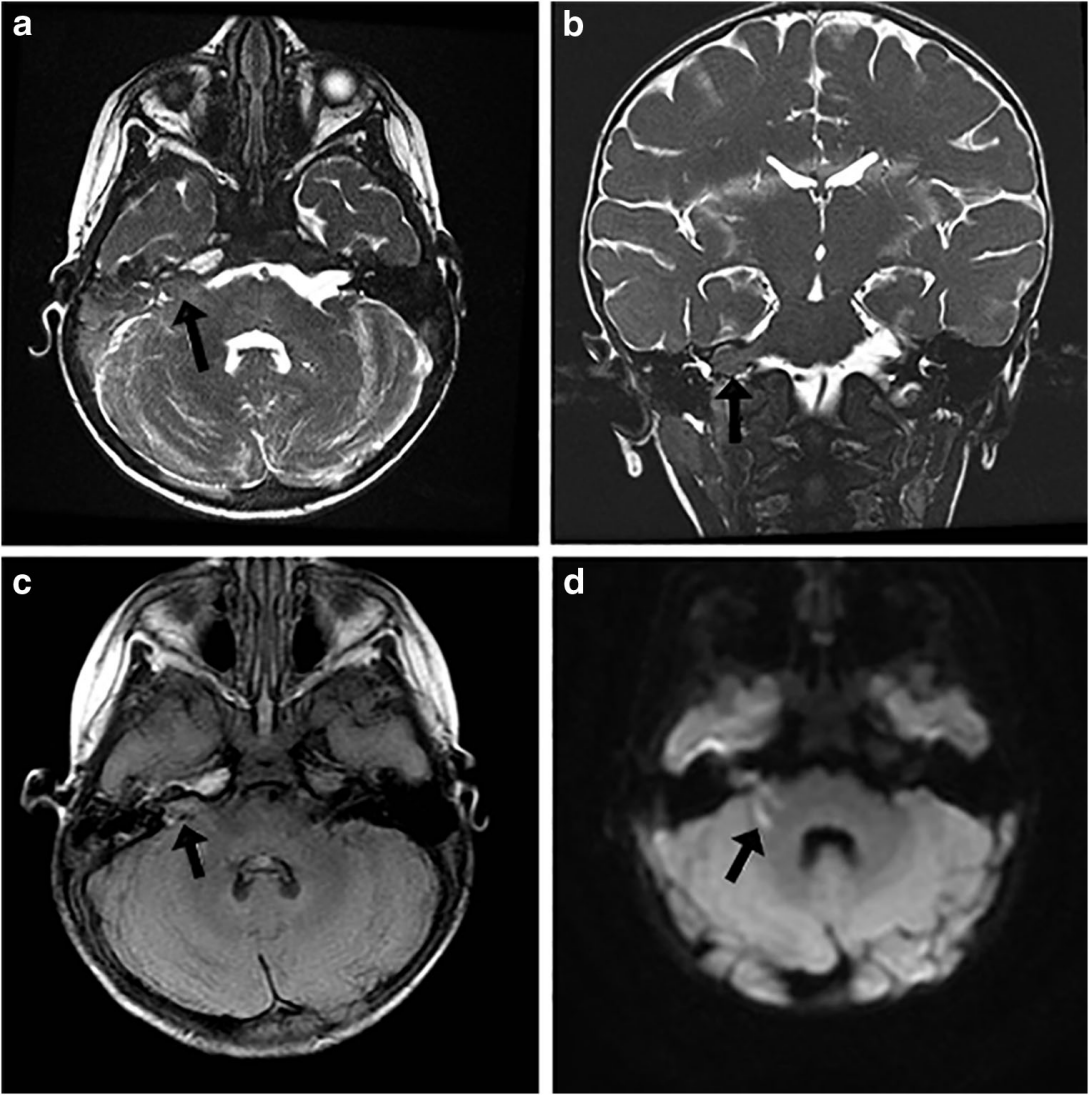
Preoperative MRI of the right cerebellopontine angle mass. **a** Axial and **b** coronal FIESTA (fast imaging employing steady-state acquisition) T2-weighted MR images showing a right cerebellopontine angle mass, with subtle T2 prolongation, inseparable with the lateral brachium pontis and extending into the internal auditory canal (arrow). **c** Axial T2 FLAIR (fluid attenuated inversion recovery) image demonstrates linear regions of T2 hyperintensity (arrow). **d** Axial diffusion-weighted image (DWI) shows linear regions of DWI hyperintensity (arrow) within the lesion (upon analysis of the apparent diffusions coefficient maps), including the component extending into the right IAC

**Fig. 2:**
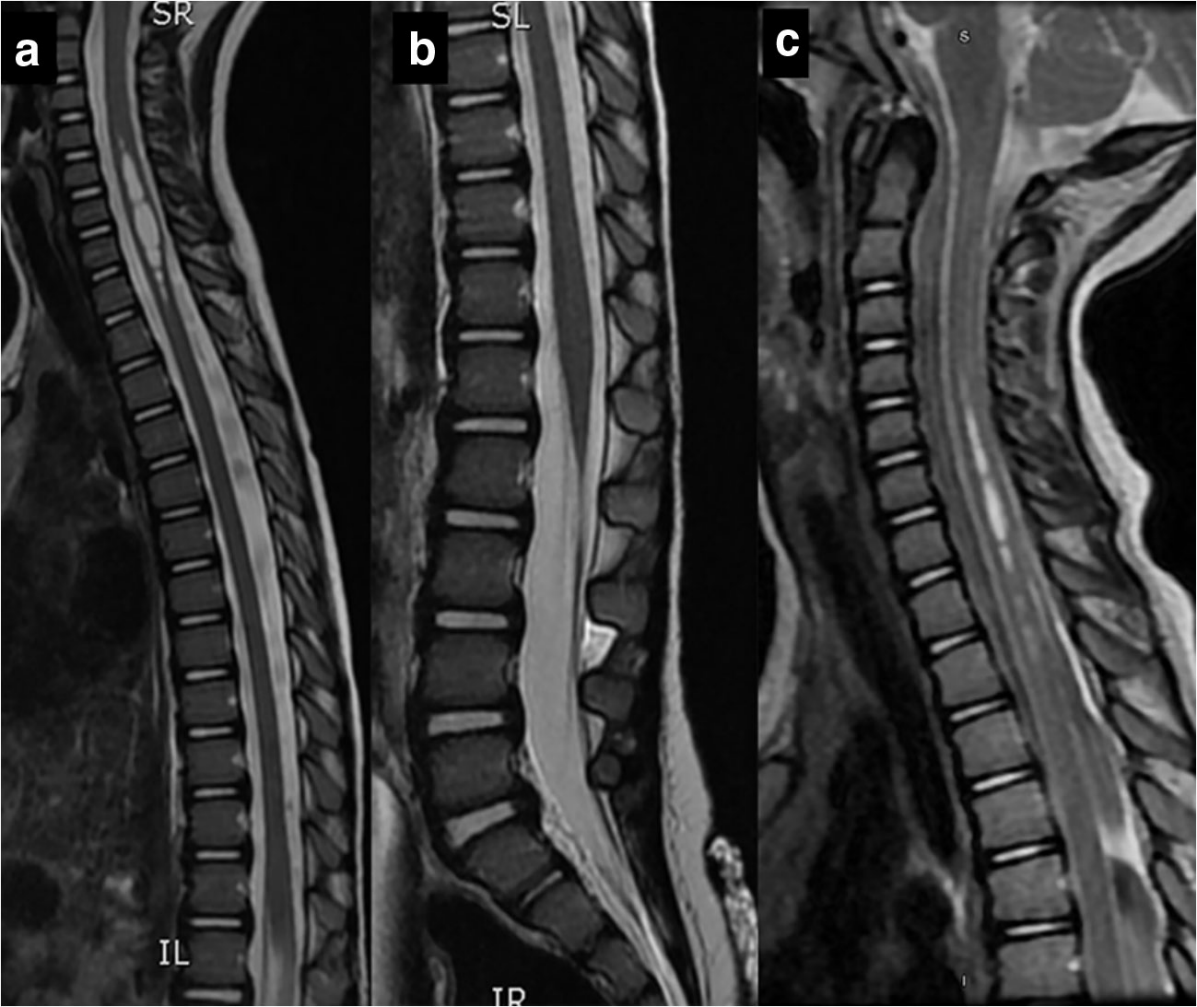
T2 cervicothoracic and lumbar MRI. **a** Cervicothoracic and **b** lumbar MRI did not demonstrate any additional lesions or tethering but did reveal cervicothoracic syrinx. **c** Cervicothoracic MRI demonstrated that the syrinx had diminished in size at two-year follow-up

A 2-year-old girl presented with worsening lethargy and a new left 6th CN palsy over 2 weeks. Her presentation prompted her physician to obtain magnetic resonance imaging (MRI) of her brain with and without contrast enhancement. T2-weighted MRI demonstrated a predominantly isointense lesion (compared with cerebellar gray matter) measuring 11 × 8 × 6 mm, centered within the right CPA and extending into the internal auditory canal (IAC) (Fig. 1a, b). It showed contiguity with the right middle cerebellar peduncle and trace contrast enhancement and linear areas of FLAIR signal hyperintensity (Fig. 1c) with corresponding areas of diffusion restriction (Fig. 1d). T2-weighted cervicothoracic and lumbar MRI were obtained (Fig. 2). A cervical syrinx was present (Fig. 2a). Despite the lack of significant enhancement, given her age, a malignant embryonal tumor such as atypical teratoid–rhabdoid tumor (ATRT) was considered. A lumbar puncture revealed an opening pressure of 36 cm H_2_O and negative cytology. She was started on dexamethasone (2 mg) every 6 h for presumed vasogenic edema. Surgery was performed to remove the mass.

For surgery, the patient was placed in left lateral position and a right retrosigmoid craniotomy was performed to access the CPA. Upon visualization, the lesion emanated directly from the anterior middle cerebellar peduncle. It then traversed the CPA and extended into the IAC lateral to the CNs. Additional drilling of the petrous bone just lateral to the IAC overlying the distal extent of the lesion was performed to expose it. The portion of the lesion extending to the IAC appeared similar to the middle cerebellar peduncle, whereas the mass within the petrous bone was not covered by dura and had a more gray appearance. The proximal base of the lesion was separated microsurgically from the middle cerebellar peduncle. The lesion within the IAC was not adherent to the 7/8 nerve complex and was removed en bloc. The dura was closed primarily and the bone plate reattached. The patient made an uneventful recovery and was weaned from medications. MRI at 1 year revealed no residual or recurrent lesion. The syrinx, which had significantly reduced in size on initial postoperative MRI, remained reduced in size (Fig. 2c). At 2-year follow-up, all preoperative symptoms had resolved.

**Fig. 3:**
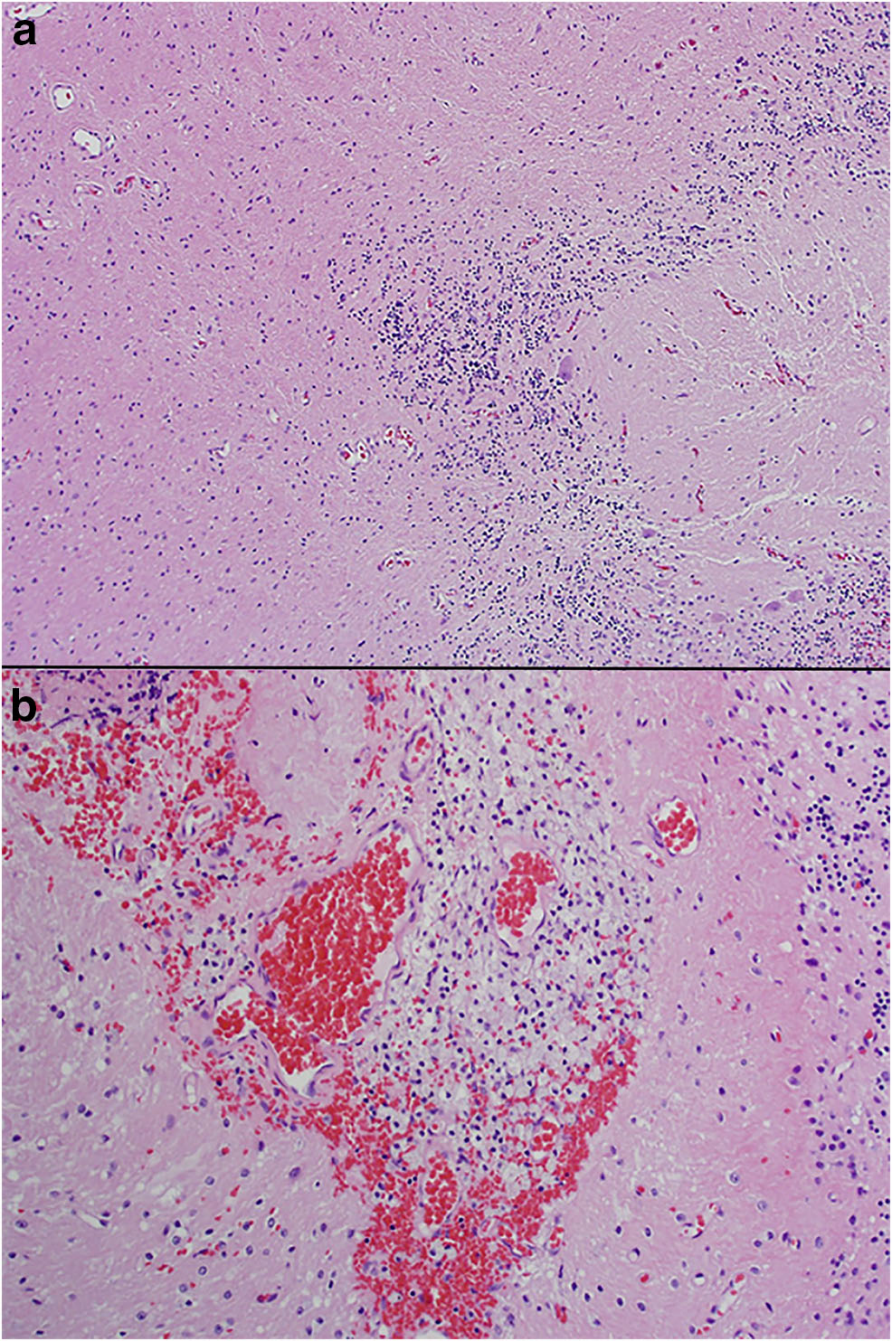
Pathology slides. **a** Histologic sections demonstrate atrophic cerebellum with focal Purkinje cell loss and Bergmann gliosis. A neoplastic glial process is not identified. **b** Clusters of foamy histiocytes are present in the leptomeninges. This histiocytic proliferation is positive for CD68 (not shown). This finding may represent a secondary reactive process

The intraoperative frozen pathological analysis demonstrated multiple small round blue cells, leading to an initial diagnosis of malignant primary central nervous system embryonal tumor. However, further pathological analysis demonstrated that these blue cells were actually granular cells of atrophic cerebellum tissue, which also contained focal Purkinje cell loss and prominent Bergmann gliosis (Fig. 3). The leptomeninges contained clusters of foamy histiocytes with small nuclei in a predominantly perivascular distribution. Other considerations based on pathological analysis, including Erdheim-Chester disease and juvenile xanthogranuloma, were ruled out because of the intraoperative appearance. There was no significant lymphocytic, eosinophilic, or neutrophilic infiltrate, and giant cells were not present. A neoplastic glial process was not identified. BRAF V600E evaluation was also negative. Follow-up S100 and CD1a stains were negative. A final pathologic diagnosis of marked cerebellar gliosis and meningeal histiocytic proliferation was given.

## Discussion

Lesions arising from the CPA with extension into the IAC in pediatric patients may include schwannomas, meningiomas, epidermoid, and arachnoid cysts, ATRT and other malignant rhabdoid tumors, or sarcomas. Meningiomas here may present with hearing loss and facial nerve palsies [3]; however, our patient’s age and lack of significant contrast enhancement on T1 imaging made meningioma less likely. The patient presentation differed from common CPA lesion mimics in key characteristics, including that the lesion was contralateral to the affected 6th nerve and not compressing it. This counterintuitive finding may be best explained by the false localization sign often associated with 6th CN palsies and was likely related to elevated intracranial pressure (ICP) [4]. Alternatively, tethering from the lesion could have stretched the contralateral 6th nerve, causing the palsy. Tethering and high ICP can lead to syrinx, which was also seen here and relieved after the surgery. Either or both processes could have led to this patient’s presentation.

The off-midline location, MRI features, and patient age suggested an aggressive lesion, possibly ATRT [5–7]. Further, the initial frozen intraoperative pathologic findings suggested a small round blue cell neoplasm. However, the formal pathological analysis revealed heterotopic non-neoplastic cerebellar tissue, and the small round blue cells were attributed to cerebellar granular cells.

The histological characteristics also suggested histiocytic disorders, but the patient lacked clinical evidence of these disorders, and the negative S100 and CD1a stains excluded a primary histiocytic process. More likely, the leptomeningeal histiocytosis was secondary to the inflammation caused by the infarction. The differentiated non-neoplastic cerebellar tissue emanating into the temporal skull base and exiting the dura defines an encephalocele; however, the tissue within was heterotopic by radiographic and hiostopathological observation.

Defining this lesion as a heterotopia would also be reasonable because cerebellar tissue does not belong within the petrous bone. Some instances of glioneuronal heterotopia are asymptomatic and discovered incidentally [8], but our patient was symptomatic upon presentation. Although heterotopias rarely grow, this lesion was most likely present at birth and became symptomatic as it grew or became tethered as the patient grew. Either process could lead to reduced perfusion and subsequent infarction, considering the small confines of the IAC and its continuity with the middle cerebellar peduncle. The pathogenesis for this glioneuronal heterotopia is likely tied to abnormal cell motility or impaired cell adhesion, leading to mislocated tissue during embryological development [9].

## Conclusion

This case represents the first heterotopic cerebellar encephalocele described within the petrous skull base. Encephaloceles containing heterotopic cerebellar tissue have been described in the anterior cranial fossa and posterior fossa [10–12]. To the best of our knowledge, encephalocele arising from the middle cerebellar peduncle and invading the IAC in a pediatric patient does not present similarly to the previously reported encephaloceles. The involvement of cranial nerves and lethargy suggested a malignant lesion; however, the intraoperative appearance outside the dura and connected directly to the brainstem, along with histological preparations that clearly revealed a nonmalignant growth with normal stromal tissue and neuropil, made this lesion consistent with the diagnosis of encephalocele. Careful assessment of the patient’s clinical presentation, detailed neurological examination, and MRI along with the gross and histological appearance are vital to correctly differentiating among various CPA lesions.
